# Development and validation of a multiplex PCR assay with melt curve analysis for detecting simian *Plasmodium* in wild *Macaca fascicularis*

**DOI:** 10.1038/s41598-025-07337-3

**Published:** 2025-07-02

**Authors:** Thitiluck Swangsri, Rucksak Rucksaken, Wanat Sricharern, Wang Nguitragool, Naowarat Saralamba

**Affiliations:** 1 Department of Molecular Tropical Medicine and Genetics, Faculty of Tropical Medicine, Mahidol University, Bangkok, 10400 Thailand; 2 Department of Veterinary Nursing, Faculty of Veterinary Technology, Kasetsart University, Bangkok, 10900 Thailand

**Keywords:** Multiplex PCR, Real-time PCR, *Plasmodium*, *Plasmodium knowlesi*, *Plasmodium cynomolgi*, *Macaca fascicularis*, Biological techniques, Medical research, Molecular medicine

## Abstract

Accurate and sensitive detection of simian malaria parasites is essential for surveillance and risk assessment of zoonotic malaria. We developed and validated a SYBR Green-based real-time PCR assay targeting the *msp1* gene to detect and differentiate *P. knowlesi, P. cynomolgi*, and *P. inui*. Species-specific amplification was confirmed through distinct melting temperature (Tm) profiles. The assay demonstrated high analytical sensitivity, with a limit of detection of 10 copies/µL, excellent specificity with no cross-reactivity, and strong reproducibility, with low coefficients of variation for both cycle threshold (Ct) and Tm values. Amplification efficiency was within acceptable ranges, with R^2^ > 0.90 across standard curves. The assay was evaluated using 191 archived blood samples from wild *M. fascicularis* collected across three provinces in Thailand, with *P. knowlesi* detected in two samples. Both positive detections were confirmed by nested PCR and sequencing. This assay offers a rapid, cost-effective, and reliable tool for detecting simian malaria parasites in laboratory analyses and has potential for further application in field surveillance.

## Introduction

Malaria remains a significant global health concern, causing substantial morbidity and mortality reported across endemic regions worldwide^1^. While human malaria has been the focus of control efforts, increasing evidence highlights the role of non-human primates (NHPs) as natural reservoirs for *Plasmodium* species capable of infecting humans^2–4^. Asymptomatic infections in NHPs, particularly in areas where humans and wildlife interact closely, contribute to the complex epidemiology of malaria and pose a risk of zoonotic transmission. Among simian *Plasmodium* species*, Plasmodium knowlesi, P. cynomolgi,* and *P. inui* are of particular concern due to their potential for cross-species transmission. Notably, *P. knowlesi* has been identified as a significant cause of human malaria in Southeast Asia, with numerous cases attributed to natural transmission from macaques to humans via mosquito vectors^5–8^. This raises concerns about the possible spillover of other simian *Plasmodium* species into human populations, emphasizing the need for enhanced surveillance and accurate diagnostic tools.

The long-tailed macaque (*Macaca fascicularis*) is a widely distributed NHP species in Southeast Asia and serves as a natural reservoir for many simian *Plasmodium* species, including *P. knowlesi*, *P. cynomolgi*, and *P. inui*^8–10^. Among these, *P. knowlesi* has been established as a zoonotic agent responsible for naturally acquired human infections, with increasing incidence reported in Malaysia and neighboring countries^8–10^. While *P. cynomolgi* and *P. inui* have historically been regarded as non-zoonotic, naturally acquired human infections with these species have been confirmed^4,11,12^, suggesting their potential for future zoonotic spillover. Recent molecular surveys have revealed high prevalence rates of *P. inui* and *P. cynomolgi* in wild *M. fascicularis* populations, frequently exceeding 50%, along with common co-infections involving multiple *Plasmodium* species^9,13,14^. In contrast, *P. knowlesi* prevalence is more variable and tends to co-circulate with other simian species^14^. These findings highlight the importance of *M. fascicularis* as a key reservoir in the ecology of simian malaria and emphasize the growing need to monitor these parasites, especially in areas where human-primate interactions are intensifying due to deforestation, agriculture, and urban encroachment. Therefore, the development and application of sensitive and specific diagnostic tools capable of accurately detecting and differentiating among *Plasmodium* species are critical. Such tools are essential not only for clinical diagnosis but also for ecological surveillance and the implementation of effective control strategies to mitigate emerging zoonotic malaria threats.

To overcome these limitations, we developed and validated a multiplex PCR assay with melt curve analysis for the simultaneous detection of *P. knowlesi, P. cynomolgi*, and *P. inui* in wild *M. fascicularis*. The *msp1* gene was selected as the target due to its species-specific sequence variability, which enables accurate discrimination among closely related *Plasmodium* species. In addition, *msp1* is a well-characterized and widely utilized genetic marker in malaria diagnostics. Its sufficient genetic divergence across simian *Plasmodium* species facilitates the design of species-specific primers, thereby supporting reliable detection and differentiation. This molecular method enables accurate and efficient detection of both single and mixed-species infections in a single reaction, improving the reliability of field diagnostics. Our approach contributes to a better understanding of simian malaria transmission dynamics and supports integrated strategies for public health monitoring and wildlife disease management^15,16^.

## Results

### PCR amplification and melt curve analysis of single-plex assays

**Fig. 1:**
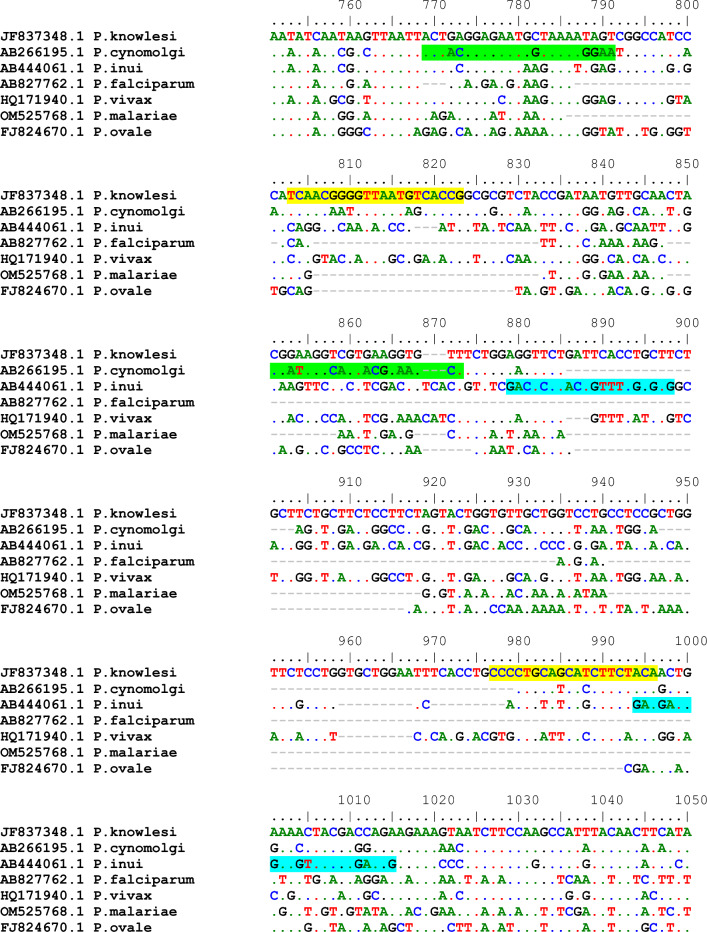
Multiple sequence alignment of *msp1* gene from *P. knowlesi* (JF37348), *P. cynomolgi* (AB266195), *P. inui* (AB444061), *P. falciparum*, *P. vivax*, *P. malariae* and *P. ovale*. The primers specific for amplification of *P. knowlesi, P. cynomolgi*, and *P. inui* were designed at the highlighted regions.

**Fig. 2:**
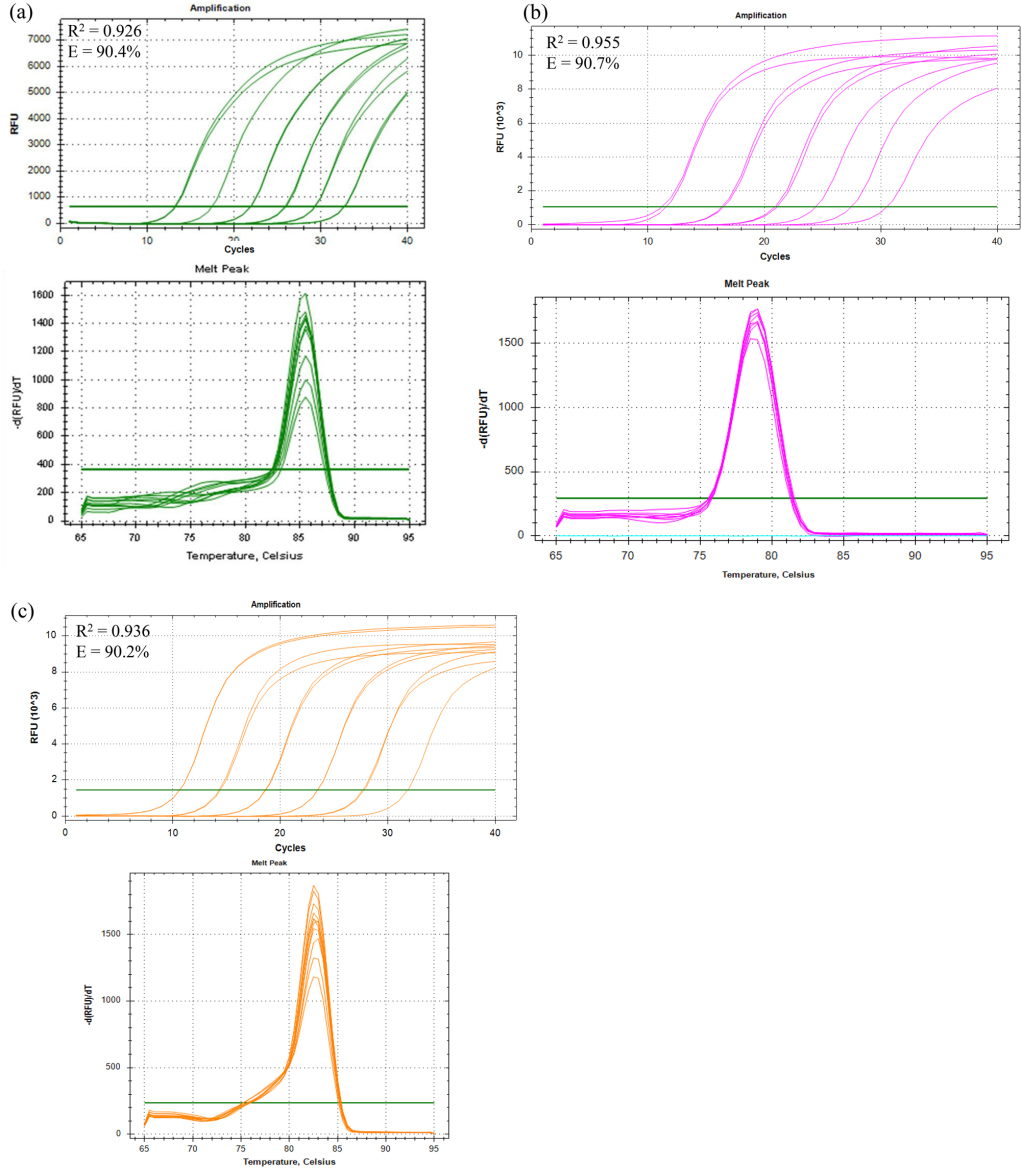
Characterization of *msp1* gene amplification using 10-fold serial dilutions from 10 to 10^6^ copies per reaction. Amplification and melt curve profiles are shown for *P. knowlesi* (**a**), *P. cynomolgi* (**b**), and *P. inui* (**c**). Each panel includes the standard amplification curve with corresponding melt curve for the respective species. Each amplification plot also displays the coefficient of determination (R^2^) and amplification efficiency (E), calculated by linear regression of the threshold cycle (Ct) values against the logarithm of the template concentrations.

Primers targeting the *msp1* gene of *P. knowlesi*, *P. cynomolgi*, and *P. inui* were designed based on sequence regions specific to each species, with alignment performed against other *Plasmodium* species known to infect humans to ensure specificity (Fig. 1). The single-plex real-time PCR assays successfully amplified the *msp1* gene fragments of *P. knowlesi, P. cynomolgi,* and *P. inui*, each yielding species-specific melting temperatures (Tm). Melt curve analysis produced distinct Tm values for each target species: *P. knowlesi* at 85.2 °C, *P. cynomolgi* at 78.0 °C, and *P. inui* at 82.5 °C (Fig. 2). No amplification was observed in non-template controls (NTCs) or in reactions containing non-target *Plasmodium* species, confirming the high specificity of each primer set.

### Sensitivity and limit of detection (LOD)

The sensitivity of the assay, both in single-plex and multiplex formats, was evaluated using 10-fold serial dilutions of plasmid DNA carrying the *msp1* gene for each *Plasmodium* species. To determine the LOD, each dilution was tested in 20 replicate reactions. The LOD was defined as the lowest DNA concentration at which 100% of replicates produced detectable amplification with a distinct species-specific melt curve peak. The assay consistently detected as few as 10 copies per reaction for *P. knowlesi*, *P. cynomolgi*, and *P. inui* across all replicates in both assay formats, demonstrating high analytical sensitivity.

### Specificity of the assay

Primer specificity was verified using genomic DNA from *P. knowlesi, P. cynomolgi*, and *P. inui*, as well as non-target *Plasmodium* species, macaque DNA, and human DNA. Each primer set amplified only its corresponding species, with no detectable cross-reactivity in any of the control reactions. This confirmed the assay’s species-level specificity.

### Reproducibility and precision

Reproducibility was assessed through intra- and inter-assay experiments. Triplicate reactions were performed using the same plasmid DNA samples at concentrations ranging from 10^2^ to 10^5^ copies per reaction, across two independent runs over a three-day period. For all three species, the mean Tm values were highly consistent, with minimal standard deviations (±0.29  °C) and low coefficients of variation (CVs ranging from 0.34 to 0.37%), indicating excellent reproducibility of species-specific melt peaks. The Ct values also demonstrated strong consistency, with intra-assay CVs ranging from 0.13 to 0.44%, and inter-assay CVs from 0.28 to 0.85%. Notably, *P. knowlesi* showed identical Tm and Ct values across both intra- and inter-assay analyses, whereas *P. cynomolgi* and *P. inui* exhibited minor variations in Ct and Tm values between runs. These findings confirm the assay’s robustness and precision for reliable detection and differentiation of the three *Plasmodium* species (Table 1).

**Table 1.** Intra- and inter-assay reproducibility of the real-time PCR assay for detection of *P. knowlesi, P. cynomolgi,* and *P. inui.*

Target species	Assay type	Mean Tm ± SD (°C)	CV for Tm (%)	Mean Ct ± SD	CV for Ct (%)
*P. knowlesi*	Intra-assay	85.16 ± 0.29	0.34	20.41 ± 0.03	0.13
	Inter-assay	85.16 ± 0.29	0.34	20.41 ± 0.10	0.47
*P. cynomolgi*	Intra-assay	78.17 ± 0.29	0.37	26.55 ± 0.12	0.44
	Inter-assay	78.33 ± 0.29	0.37	25.53 ± 0.07	0.28
*P. inui*	Intra-assay	82.83 ± 0.29	0.35	25.35 ± 0.08	0.30
	Inter-assay	82.83 ± 0.29	0.35	26.21 ± 0.22	0.85

### Multiplex performance and detection of mixed infections

**Fig. 3:**
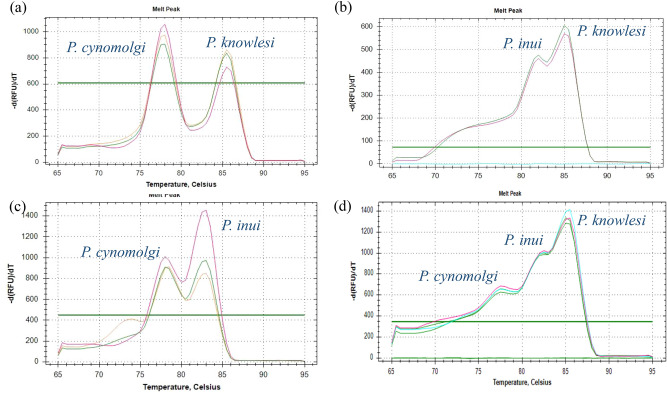
Differentiation of mixed infections using melting peak analysis. Melting curve profile are shown for samples containing mixed infection of (**a**) *P. knowlesi* + *P. cynomolgi,* (**b**) *P. knowlesi* + *P. inui*, (**c**) *P. cynomolgi* + *P. inui,* and (**d**) *P. knowlesi* + *P. cynomolgi* +*P. inui*. Each colored line in the graphs represents an individual sample.

In the multiplex PCR assay, all three species were simultaneously detected in a single reaction. Artificially prepared DNA mixtures containing two or three *Plasmodium* species, with varying concentrations ranging from 10^2^ to 10^4^ copies per reaction in different combinations, generated multiple, well-resolved melt peaks corresponding to each target species. Amplification and melt curve profiles were consistent with those obtained in the single-plex reactions, with clearly resolved Tm peaks for each species (Fig. 3). No cross-reactivity, nonspecific amplification, or primer-dimer formation was observed. Additionally, there was no overlap or distortion of the melt profiles, confirming that the assay can reliably discriminate between single and mixed-species infections based on distinct melting temperature profiles.

### Application of the assay to archived field samples

**Fig. 4:**
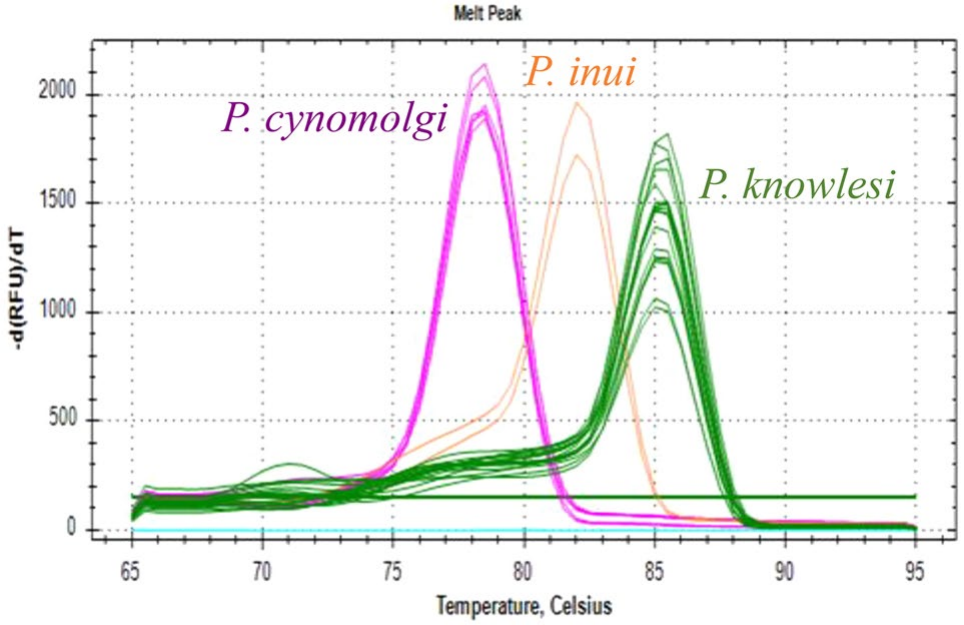
Melt peak profiles of positive field samples re-analyzed using species-specific real-time PCR melt curve analysis, alongside known positive controls (8 *P. knowlesi* 3 *P. cynomolgi*, and 1 *P. inui*). Distinct melting peaks corresponding to *P. knowlesi* (85.2 °C, green), *P. cynomolgi* (78 °C, magenta), and *P. inui* (82.5 °C, orange) are observed in the control samples. The two field samples exhibited clear melt peaks aligning with *P. knowlesi*, confirming their positive status upon repeat testing.

A total of 191 archived blood samples from wild *M. fascicularis* were screened using the developed multiplex, species-specific real-time PCR melt curve assay. The samples originated from three locations: Prachuap Khiri Khan (n = 66), Songkhla (n = 39), and Lopburi (n = 86). In parallel, all samples were also tested using genus-specific nested PCR targeting *Plasmodium* species. Two samples, one from Songkhla and one from Lopburi, tested positive by both methods. These two samples exhibited distinct melt peaks at 85.2  °C, aligning with *P. knowlesi*, and were further confirmed by sequencing of the nested PCR products. To validate the melt curve assay, the two positive samples were re-analyzed alongside known *P. knowlesi*, *P. cynomolgi*, and *P. inui* positive controls, showing consistent melt peak profiles (Fig. 4). No *P. cynomolgi* or *P. inui* infections were detected among the field samples. The results demonstrate complete concordance between the real-time PCR melt curve assay, genus-specific nested PCR and sequencing, confirming the accuracy and reliability of the method.

## Discussion

In this study, we successfully developed and validated a SYBR Green-based multiplex real-time PCR assay with melt curve analysis for the simultaneous detection of *P. knowlesi, P. cynomolgi*, and *P. inui* in wild *M. fascicularis*. This assay represents a significant advancement over conventional diagnostic techniques by enabling the detection of multiple *Plasmodium* species in a single closed-tube reaction. Compared to nested PCR, which is widely used but time-consuming, labor-intensive, and prone to contamination due to its multi-step workflow^17,18^, our melt curve-based assay offers a faster, simpler, and more contamination-resistant alternative. While microscopy remains the traditional gold standard, it lacks the sensitivity to detect low parasitemia or mixed-species infections and requires considerable expertise for accurate species identification^19^. Our assay demonstrated high analytical sensitivity, specificity and reproducibility. Moreover, it offers a relatively cost-efficient alternative to probe-based or nested PCR assays, despite the absence of a formal cost-effectiveness analysis. The use of SYBR Green eliminates the need for expensive fluorescent probes, and the single-reaction format reduces reagent usage, labor, and hands-on time. The protocol is compatible with standard real-time PCR platforms commonly available in diagnostic and research laboratories, making it particularly suitable for routine application in endemic settings and for large-scale ecological surveillance of simian malaria parasites.

Species differentiation in our assay was achieved via melting temperature (Tm) analysis, with reproducible Tm values for *P. knowlesi* (85.2  °C), *P. cynomolgi* (78.0 °C), and *P. inui* (82.5 °C). These melt signatures allowed for precise discrimination without the need for fluorescent probes, thereby reducing assay costs and complexity while maintaining high specificity. The assay’s compatibility with SYBR Green and a standard real-time PCR platform also supports its applicability in resource-limited settings and field laboratories^20,21^.

Importantly, the assay reliably detected mixed infections in controlled DNA mixtures, confirming its suitability for ecological and epidemiological studies where co-infections are common^9,13^. The ability to detect multiple *Plasmodium* species is critical for understanding interspecies interactions, host susceptibility, and the potential for recombination or co-transmission events, all of which play key roles in shaping zoonotic transmission dynamics. However, a potential limitation of the assay is its reduced sensitivity when one species is present at a much lower parasitemia compared to others in a mixed infection. Future optimization and testing using artificially varied ratios of parasite DNA could help assess and improve the assay’s ability to detect minor components in mixed-species infections.

Application of the assay to 191 archived blood samples from *M. fascicularis* across three provinces in Thailand identified two individuals infected with *P. knowlesi*, reinforcing the role of *M. fascicularis* as a natural reservoir for this zoonotic parasite. This finding aligns with the well-documented prevalence of *P. knowlesi* as the most frequently reported simian *Plasmodium* species infecting humans in Southeast Asia^22,23^. Notably, the detection of *P. knowlesi* in Songkhla province corresponds with previously reported human cases in the region^24^, while its identification in Lopburi, where no human infections have yet been reported, raises concerns about potential silent transmission. These observations highlight the importance of sustained surveillance, even in areas not currently considered endemic, as infections in reservoir hosts and vectors may serve as early indicators of future human outbreaks. No *P. cynomolgi* or *P. inui* infections were detected in this sample set, despite the assay’s analytical sensitivity of 10 copies/μL. This absence may reflect a combination of ecological and epidemiological factors, including temporal fluctuations in parasite prevalence, spatial heterogeneity in transmission dynamics, and complex host-parasite-vector interactions. Environmental variables likely influencing parasite distribution include vector species composition and competence, macaque population density, and habitat characteristics such as forest cover, elevation, and proximity to human settlements^9,25^. These factors collectively affect exposure risk and transmission intensity. Additionally, anthropogenic disturbances such as deforestation and land-use conversion may alter vector habitats and disrupt established transmission cycles. Importantly, the absence of *P. cynomolgi* and *P. inui* signals in both the SYBR Green-based assay and a genus-specific nested PCR assay (with a reported limit of detection of ~1–5 parasites/μL) suggests either true absence of these parasites in the sampled population or parasitemia levels below the detection thresholds of both methods. Nonetheless, the demonstrated capacity of the multiplex assay to sensitively and specifically detect all three target species reinforces its potential utility for long-term ecological surveillance, zoonotic risk assessment, and wildlife pathogen monitoring.

One limitation of the current study is the relatively low prevalence of positive samples, which restricted comprehensive evaluation of assay performance across all target species in field samples. Additionally, the assay was tested primarily on archived samples rather than freshly collected specimens, and therefore its performance under actual field conditions remains to be fully assessed. While the assay demonstrated strong analytical sensitivity and specificity in controlled laboratory settings, the limited number of naturally infected samples, particularly those positive for *P. cynomolgi* and *P. inui*, precluded any robust assessment of the assay’s diagnostic sensitivity and specificity in naturally infected field samples. Future research involving larger sample sizes, additional geographic regions, temporal monitoring, and evaluation with freshy collected field samples is essential to comprehensively assess the assay’s diagnostic sensitivity, specificity, and robustness under diverse field conditions. Moreover, integration of this assay with entomological and human surveillance data will facilitate a comprehensive understanding of zoonotic malaria transmission dynamics.

In conclusion, the multiplex real-time PCR assay with melt curve analysis developed in this study is a robust, scalable tool for detecting simian *Plasmodium* species in reservoir hosts. Its high analytical performance and capacity for detecting co-infections support its application in wildlife surveillance, ecological research, and early detection of potential zoonotic malaria outbreaks. As zoonotic malaria continues to pose a growing public health challenge in Southeast Asia, molecular tools such as this are critical for informing control strategies and safeguarding human health.

## Methods

### Samples collection and preparation

Genomic DNA from *P. knowlesi* was obtained from an *in vitro* culture maintained in the laboratory. Genomic DNA of *P. cynomolgi* and *P. inui* was purchased from the American Type Culture Collection (ATCC, USA).

Archived blood samples from *M. fascicularis* were originally collected during macaque population control programs, including sterilization campaigns, conducted by veterinary teams in collaboration with local authorities. These samples were obtained as part of a broader pathogen surveillance effort, specifically targeting *Bartonella quintana*. The current study repurposed these archived samples for molecular screening of simian *Plasmodium* species. Sampling was carried out in three provinces of Thailand: Prachuap Khiri Khan (August 2016, n = 66), Songkhla (July 2018, n = 39), and Lopburi (April 2018, n = 86). Following collection, all blood samples were preserved in EDTA tubes and immediately stored at −80  °C to maintain DNA integrity. Prior to pathogen detection, total DNA was extracted, and its quality was assessed by spectrophotometric analysis (A260/A280). In addition, the host *G6PD* gene was successfully amplified in a subset of samples to confirm host DNA integrity and validate the efficiency of the extraction process. The DNA samples also yielded successful amplification in genus-specific *Plasmodium* nested PCR assays used for method comparison, further confirming the absence of significant degradation.

All protocols related to animal handling and blood collection were reviewed and approved by the Animal Ethics Committee of Kasetsart University, Bangkok, Thailand (Approval No. ACKU59-VTN-004). The experimental procedures were also ethically approved by the Faculty of Tropical Medicine, Mahidol University (Certificate No. MUTM 2023-089-01). All methods were carried out in accordance with relevant guidelines and regulations. All methods are reported in accordance with the ARRIVE guidelines (https://arriveguidelines.org).

### Primer design

Species-specific primers targeting the *msp1* gene of *P. knowlesi, P. cynomolgi*, and *P. inui* were designed manually following multiple sequence alignment using BioEdit version 7.2.5. The designed primers were validated using NCBI Primer-BLAST to ensure species specificity. Target regions were selected based on interspecies sequence variability to ensure high specificity and maximize discriminatory power. Primer selection was guided by standard parameters, including optimal melting temperatures (58–60 °C), GC content (40–60%), and amplicon sizes suitable for SYBR Green-based melt curve analysis (<200 bp). Potential secondary structures, such as hairpins and primer-dimers, were assessed *in silico* using the OligoAnalyzer tool (Integrated DNA Technologies, https://www.idtdna.com). Figure 1 shows the primer binding sites and corresponding amplicon sizes.

### Positive control plasmids preparation and sequencing

Positive control plasmids were constructed by amplifying short fragments of the *msp1* gene from each *Plasmodium* species (see Fig. 1), followed by cloning into the pGEM®-T Easy Vector System (Promega, USA). The recombinant vectors were transformed into competent *Escherichia coli*, and positive clones were cultured. Plasmid DNA was extracted and sequenced via Sanger sequencing (U2Bio, Thailand). The identity of the inserted sequences was verified using the NCBI BLASTN tool.

### PCR and melting curve

Single-plex PCR reactions were performed in a 10 μL total volume containing 300 nM of each primer pair:

- *P. knowlesi*: Pk MSP1 F626 (5′-TCAACGGGGTTAATGTCACCG-3′) and Pk MSP1 R816 (5′-TGTAGAAGATGCTGCAGGGG-3′)
- *P. cynomolgi*: Pcy MSP1 F600 (5′-ACTACGGAGAATGGTAAAAGGAA-3′) and Pcy MSP1 R699 (5′-AGCTTCCGTACTGCCTATCG-3′)
- *P. inui*: Pin MSP1 F702 (5′-GACTCCTACTGTTTCGGGTG-3′) and Pin MSP1 R820 (5′-CCTTCTCGTAACTTCCATCTTC-3′)

Annealing temperature was initially estimated based on primer Tm values and optimized via gradient PCR (55–65  °C). An annealing temperature of 60  °C was identified as optimal and used throughout all subsequent assays.

Each reaction contained 1× iTaq Universal SYBR Green Supermix (Bio-Rad, USA) and 2 μL of DNA template. Amplification was performed using a Bio-Rad CFX Connect Real-Time PCR System with the following cycling conditions: initial denaturation at 95  °C for 5 min, followed by 40 cycles of 95  °C for 5 s and 60  °C for 30 s.

Melting curve analysis was conducted from 65 to 95  °C in 0.5  °C increments with a 2-second hold per step. Multiplex PCR reactions containing all three primer sets were conducted under the same thermal conditions as the single-plex reactions.

### Assay validation

#### Sensitivity

The analytical sensitivity of the assay was evaluated using 10-fold serial dilutions of plasmid DNA containing the *msp1* target sequence for each *Plasmodium* species. DNA concentrations ranged from 10^6^ to 1 copy/μL. To determine the limit of detection (LOD), each dilution point was tested in 20 independent replicate reactions. The LOD was defined as the lowest DNA concentration at which all 20 replicates (100%) produced positive amplification with a distinct and species-specific melt curve peak. Amplification efficiency was compared between the single-plex and multiplex formats.

#### Specificity

The specificity of each primer set was tested using genomic DNA from *P. knowlesi, P. cynomolgi,* and *P. inui.* To assess cross-reactivity, non-template controls (NTCs), DNA from unrelated *Plasmodium* species (*P. falciparum*, *P. vivax*, *P. malariae*, and *P. ovale*), macaque host DNA, and human DNA were included. Melt curve analysis confirmed species-specific amplification, with each target producing a distinct melting temperature (Tm) peaks.

#### Reproducibility

Intra-assay and inter-assay reproducibility were assessed by performing triplicate PCR reactions across different days using the same DNA samples. Coefficients of variation (CVs) were calculated for both cycle threshold (Ct) values and melting temperatures (Tm). Intra-assay variation was determined from triplicates reactions performed on the same day, while inter-assay variation was assessed from the average of reactions conducted on different days. Low CVs and consistent Tm readings across replicates indicated high precision and robustness of the assay.

#### Multiplex performance

The multiplex assay was further validated using DNA mixtures containing two or three *Plasmodium* species to evaluate the assay’s ability to detect and differentiate co-infections. Distinct melt peaks corresponding to each target species confirmed the assay’s capability to identify multiple targets in a single reaction.

### Statistical analysis

Intra-assay and inter-assay variation in the Ct values were determined from triplicate real-time PCR reactions using the same plasmid DNA samples at concentrations ranging from 10^2^ to 10^5^ copies per reaction. Intra-assay variation was assessed from triplicates performed on the same day, while inter-assay variation was evaluated by repeating the assay on a separate day (three days later). The mean threshold cycle (Ct) values and melting temperatures (Tm) were calculated along with their standard deviations (SD). To further evaluate the precision of the assay, the coefficient of variation (CV) was computed for both Ct and Tm values. Low CV values indicated high precision and consistency of the assay, both within and between runs. All statistical calculations were performed using Microsoft Excel, and data were presented as mean ± SD with corresponding CV percentages.

## Data Availability

The authors declare that the data supporting the finding in this study are available within the paper.
